# Do people reach 100 by surviving, delaying, or avoiding diseases? A life course comparison of centenarians and non-centenarians from the same birth cohorts

**DOI:** 10.1007/s11357-024-01330-w

**Published:** 2024-08-30

**Authors:** Yuge Zhang, Shunsuke Murata, Katharina Schmidt-Mende, Marcus Ebeling, Karin Modig

**Affiliations:** 1 Unit of Epidemiology, Institute of Environmental Medicine, Karolinska Institutet, Box 210, 17177 Stockholm, Sweden; 2 Academic Primary Health Care Centre, Stockholm Region, Stockholm, Sweden; 3 Division of Family Medicine and Primary Care, Department of Neurobiology, Care Sciences and Society, Karolinska Institutet, Huddinge, Sweden; 4 Max Planck Institute for Demographic Research, Rostock, Germany

**Keywords:** Centenarians, Lifetime risk, Age-specific incidence rate, Birth cohort, Longevity

## Abstract

Centenarians are perceived as pioneers of longevity, possessing the secrets to surpassing age 100. It remains unclear whether they achieve this by surviving, delaying, or avoiding diseases to a greater extent than their shorter-lived peers. This register-based study encompassed all individuals aged 60 and older, born between 1912 and 1922 in Stockholm County, Sweden (*N* = 170,787). Using historical data, individuals were prospectively followed from 1972 to 2022 and stratified by their age at death. Age-specific incidence rates and remaining lifetime risk from age 60 were calculated for stroke, myocardial infarction, hip fracture, and various cancers (including colorectal, breast, and prostate), and compared between those who survived to age 100 and their shorter-lived counterparts. Centenarians had lower age-specific incidence rates for almost all diseases and ages. Despite longer life spans, their lifetime risks for all diseases except hip fracture were lower than those of non-centenarians. This suggests that centenarians delay, and even avoid, many of the major age-related diseases rather than surviving them to a higher extent. The findings that centenarians not only exhibit lower disease rates at younger ages compared to their shorter-lived peers but throughout their lives challenge the notion that longer life span inevitably leads to higher disease rates or a simple shift of diseases to older ages.

**Supplementary Information:**

The online version contains supplementary material available at 10.1007/s11357-024-01330-w.

## Introduction

Surviving to at least 100 years old is rare, yet centenarians are currently the fastest growing segment of the world’s population [1]. They are seen as pioneers of longevity, possessing the secrets to a long life. It is largely unknown if they do so by surviving disease to a higher extent than their shorter-lived peers, or if they delay or even avoid disease.

Several studies have investigated routes to achieving exceptional longevity by analyzing centenarians alone. These studies indicate that centenarians are a heterogeneous group of individuals, with different disease profiles [2–7], and far from being fully healthy. An Italian study based on 602 centenarians found that only 20% of the centenarians were in good health, 33% had intermediate status, and 47% were in poor health [2]. A German study using health insurance data from 1121 centenarians identified four distinct health profiles and only a small proportion of centenarians were healthy [7]. A few studies also found centenarians can be categorized into survivors, delayers, and escapers of diseases, but without comparing to their shorter-lived peers [3, 8]. Some previous research suggests that centenarians have a delayed onset of several diseases in comparison to non-centenarians [9–11]. On the contrary, some study described centenarians as a rather unhealthy group with declined physical abilities, polypharmacy, and pronounced care needs [12–15]. This points more towards better survival with disease rather than avoidance of disease. However, most previous studies analyzed centenarian populations without any comparison to their shorter-lived peers [4, 5, 15]. Such a comparison would be necessary to investigate if centenarians survive, delay, or even avoid disease to a higher extent. Only a comparison to shorter-lived individuals at comparable ages earlier in life would allow us to understand this. If centenarians suffer from diseases at the same rate but survive them at a higher rate, the mechanisms behind their exceptional longevity suggest that they are resilient to specific diseases and that they are susceptible to secondary prevention and treatment. If centenarians delay or avoid diseases, it rather points towards centenarians being more resilient to the disease itself, perhaps through more favorable lifestyle factors, perhaps through genetic factors.

Additional constraints within the current body of literature that use centenarians to explore longevity include cross-sectional designs [3, 13], which excludes the possibility to compare disease onset, the use of self-reported information on disease history which potentially introduces recall bias [6, 8], and the analysis of health selected samples where institutionalized individuals are excluded, or non-representative data sources for example health insurance data [10, 11, 14] which potentially skew the centenarians’ health status towards healthier than it is. Finally, cohort effects could distort the comparison of centenarians with shorter lived individuals when compared in a cross-sectional way [9, 10]. Ideally, subjects born at the same time as centenarians should be used for comparison, but such longitudinal data are scarce.

In this study, we explored how disease onset and disease accumulation differ across the life course between centenarians and non-centenarians by utilizing historical but prospective data from population-wide administrative health records. The aim was to explore whether centenarians reach age 100 by surviving, delaying or avoiding diseases by comparing age-specific incidence rates and lifetime risk of different diseases from age 60 and onwards among individuals reaching 100 years, and their shorter-lived peers born during the same years.

## Methods

### Data source and study population

The study population includes individuals from birth cohorts 1912 to 1922, who were residing in Stockholm County from age 60 onward. Individuals were followed prospectively in the registers from January 1, 1972, until death, their 100th birthday, or censoring on December 31, 2022. This enabled follow-up of all individuals from age 60 to 100.

### Ascertainment of diseases

Through a unique personal identification number, information about diseases and causes of death was linked to each individual from the National Patient Register [16], the National Cancer Register [17], and the Cause of Death Register [18]. Included diseases were stroke, myocardial infarction (MI), hip fracture, and any cancer. These were chosen because they are age-related diseases and account for a large part of the disease panorama in the population [19]. They are also well-suited for identification in the included registers because they require hospitalization, have a clear onset, and are mostly accurately diagnosed [16, 19, 20]. Any cancer was additionally specified into the most common cancer types, colorectal cancer, breast cancer (women only), and prostate cancer (men only) [21]. Myocardial infarction, stroke, and hip fracture were identified through hospitalization with a corresponding main diagnosis code. Cancer was identified through the Cancer register. Diagnoses were identified using codes from the International Classification of Diseases (ICD) system versions 7 to 10. The following ICD codes were used to identify main diagnosis for respective disease of interest: stroke 430–34,436 (ICD-8), 430–34,436 (ICD-9), I60-I64 (ICD-10); MI 410 (ICD-8), 410 (ICD-9), I21-22 (ICD-10); hip fracture 820 (ICD-8), 820 (ICD-9), S72.0-S72.2 (ICD-10); cancer 140–209 (ICD-7), 140–209 (ICD-9), C00-C95 (ICD-10); colorectal cancer 153, 154.0 (ICD-7), 153, 154.0, 154.1 (ICD-9), C18-C20 (ICD-10); breast cancer 170 (ICD-7), 174 (ICD-9), C50 (ICD-10); and prostate cancer 177 (ICD-7), 185.9 (ICD-9), C61 (ICD-10).

Education level, defined as a person’s highest achieved level of education, was obtained from Population and Housing Census 1970 and described for centenarians and non-centenarians because of its association with health-related outcomes and other socioeconomic factors [22]. Level of education was classified into low (compulsory, up to 8–9 years), mid (upper secondary, up to 2–3 years), and high (undergraduate or graduate level).

### Statistical analysis

Age-specific incidence rates of the respective disease were calculated from age 60 and onwards and presented for individuals based on their age at death. All individuals aged 60 years and above were included, regardless of the age when they entered the cohort after their 60th birthday (an open cohort design). 5.2% of individuals immigrated into the study population after age 60. For MI and stroke, all incident cases since age 60 were included. An incident case was defined as a case occurring at least 28 days apart from a previous one as applied by the Swedish National Board of Health and Welfare [23, 24]. For cancer and hip fracture, only the first case since age 60 was considered since attaining more than one primary tumor or hip fracture is rare. Incidence rates were calculated as the number of disease events at each age divided by the corresponding person time at risk. To reduce random fluctuations that result from small number of cases at younger ages, we smoothed the incidence rates by using P-splines within a Poisson generalized additive model with person time as offset. Percentile bootstrap methods based on 1000 iterations were applied to estimate 95% confidence intervals (95% CIs) of the incidence rates. The last year of life was excluded from the fitting to avoid overestimation of incidence rates due to the impact of individuals dying from the disease. Observed incidence rates were also presented in supplementary materials to show the consistency between smoothed and observed rates.

To understand how the risk of being diagnosed with a certain disease varies over lifespan length, we additionally calculated the cumulative incidence considering only the first event. This was done by taking the number of disease events occurring at each age divided by the number of individuals at baseline (age 60, a closed cohort design), stratified by age-at-death in 10-year age groups. 95% CIs of cumulative incidence were estimated with Clopper-Pearson method. The analyses present the proportion of centenarians and non-centenarians that were diagnosed with a respective disease at a given age. Since we followed the individuals until they died or reached age 100, the cumulative incidence can be interpreted as the remaining lifetime risk from age 60 of the respective disease. Sex-specific analyses were additionally estimated and presented as supplementary results.

All statistical analyses were conducted using SAS 9.4 (SAS Institute Inc., Cary, NC, USA) and R (version 4.1.2; R Foundation for Statistical Computing, Vienna, Austria).

## Results

Table 1 provides a description of the study population stratified by age at death in 10-year age groups. A total of 170,787 individuals were included, of whom 52.8% were women and 47.2% were men. Among the 170,787 individuals, 1.4% became centenarians, 20.0% died between ages 90–99 years, 35.0% died between 80 and 89 years, 26.9% died between 70 and 79 years, and 16.7% died between 60 and 69 years. The proportion of low education was higher among individuals dying before average life expectancy than among individuals reaching ages above 90 years. For ages above 90, the level of education was very similar. Approximately 23.6% had at least one stroke during the follow-up, 23.5% had at least one MI, 18.0% had a hip fracture, 29.7% had any type of cancer, 4.9% colorectal cancer, 3.8% breast cancer, and 5.3% prostate cancer. The median ages at diagnosis, except for hip fracture at 82.0 years, were in participants’ 70 s. The age distribution of disease onset is shown in Supplementary Table S3. Over 60% of people were diagnosed in their 70 s and 80 s. Sex-specific results are shown in Supplementary Table S1-2 and Table S4-5. More men than women died before age 80 and men had a much smaller chance of becoming a centenarian, 0.5% compared to 2.3% for women. Men also had a higher share of MI and cancer, and a younger age at onset for all included diseases.

**Table 1:** Characteristics of study participants, total and stratified by age at death, aged 60 years and above in Stockholm County, Sweden

	Died between ages 60–69 years	Died between ages 70–79 years	Died between ages 80–89 years	Died between ages 90–99 years	Died ≥ 100 years	Total	Age at diagnosis, median (IQR) ^a^
No. of individuals, (%)	28,487 (16.7)	45,876 (26.9)	59,830 (35.0)	34,227 (20.0)	2367 (1.4)	170,787	
No. of female, (%)	10,120 (35.5)	20,050 (43.7)	33,990 (56.8)	24,019 (70.2)	1989 (84.0)	90,168 (52.8)	
No. of education^b^, (%)							
Low	16,050 (56.3)	26,324 (57.4)	33,729 (56.4)	18,343 (53.6)	1256 (53.1)	95,702 (56.0)	
Mid	7905 (27.8)	13,046 (28.4)	17,933 (30.0)	10,953 (32.0)	749 (31.6)	50,586 (29.6)	
High	1871 (6.6)	3554 (7.8)	5337 (8.9)	3718 (10.9)	282 (11.9)	14,762 (8.6)	
Missing	2661 (9.3)	2952 (6.4)	2831 (4.7)	1213 (3.5)	80 (3.4)	9737 (5.7)	
No. of patients (%)							
Stroke	3063 (10.8)	9740 (21.2)	17,329 (29.0)	9723 (28.4)	439 (18.5)	40,294 (23.6)	79.0 (72.0–85.0)
Myocardial infarction	6462 (22.7)	11,762 (25.6)	14,560 (24.3)	6997 (20.4)	295 (12.5)	40,076 (23.5)	76.0 (69.0–83.0)
Hip fracture	933 (3.3)	4610 (10.0)	13,247 (22.1)	11,097 (32.4)	836 (35.3)	30,723 (18.0)	82.0 (76.0–88.0)
All cancer	9136 (32.1)	16,439 (35.8)	17,952 (30.0)	9175 (26.8)	608 (25.7)	53,310 (29.7)	74.0 (68.0–81.0)
Colorectal cancer	1110 (3.9)	2226 (4.9)	3124 (5.2)	1758 (5.1)	115 (4.9)	8333 (4.9)	76.0 (70.0–83.0)
Breast cancer	528 (1.9)	1520 (3.3)	2478 (4.1)	1798 (5.3)	139 (5.9)	6463 (3.8)	74.0 (67.0–81.0)
Prostate cancer	631 (2.2)	2649 (5.8)	4081 (6.8)	1590 (4.6)	36 (1.5)	8987 (5.3)	76.0 (71.0–81.0)

^a^*IQR* interquartile range

^b^For education level, low represents compulsory level (up to 8–9 years), mid represents upper secondary level (up to 2–3 years), and high represents undergraduate or graduate level

**Fig. 1:**
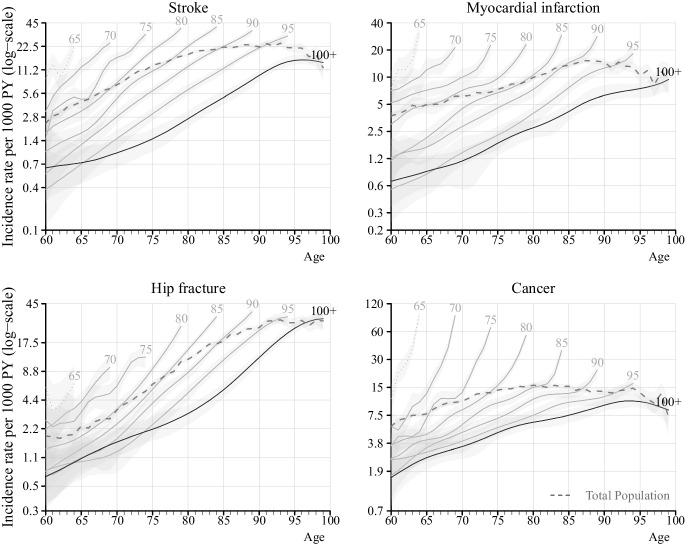
Age-specific incidence rates of stroke, myocardial infarction, hip fractures, and cancer from age 60 for individuals born between 1912 and 1922, by age at death, in Stockholm County, Sweden. Notes: Solid lines represent the smoothed rates while dashed line for age 65 represents the observed rates. The light grey area represents the 95% confidence interval. The *x*-axis represents the chronological ages of everyone followed from age 60 until death or becoming centenarians. The numbers by each line represent age at death

**Fig. 2:**
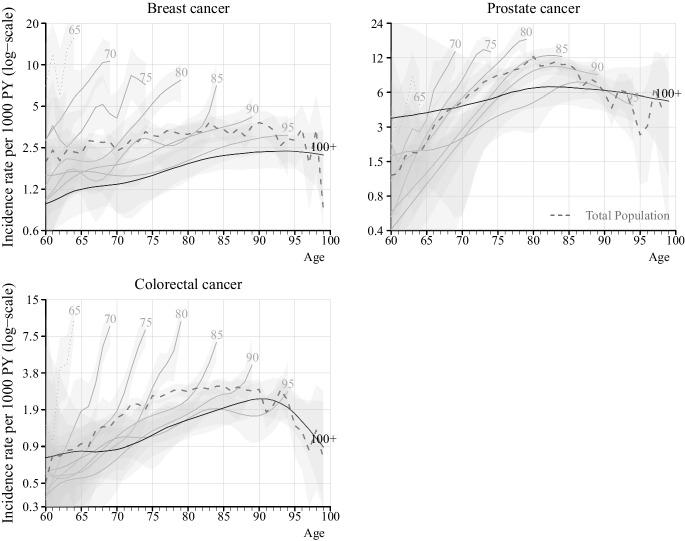
Age-specific incidence rates of breast, prostate, and colorectal cancer from age 60 for individuals born between 1912 and 1922, by age at death, in Stockholm County, Sweden. Notes: Solid lines represent the smoothed rates while dashed line for age 65 represents the observed rates. The light grey area represents the 95% confidence interval. The *x*-axis represents the chronological ages of everyone followed from age 60 until death or becoming centenarians. The numbers by each line represent age at death

Figures 1 and 2 illustrate the age-specific incidence rates of respective disease from age 60 and onwards for different ages at death. For the total population, with the exception of hip fracture, incidence rates increased exponentially with age, followed by a decline at age 87 for MI, age 91 for stroke, and age 80 for cancer. Notably, the incidence rate at age 60 was higher among individuals who died at younger ages. Age-specific incidence rates were highest among those who died earlier and lowest among those who lived longer, with centenarians exhibiting the lowest rates. When compared to non-centenarians, centenarians consistently had the lowest incidence rates across nearly all ages and diseases. For colorectal and prostate cancer, this difference was particularly pronounced up to individuals dying at age 90.

Although the 95% confidence intervals (CIs) are wide at younger ages due to the small number of cases, differences at higher ages with non-overlapping CIs can be observed between centenarians and non-centenarians for stroke, MI, hip fractures, and various cancers. For specific cancer sites, the small number of cases results in wide and overlapping 95% CIs; nonetheless, the pattern of lower incidence rates among centenarians remains evident. This trend of centenarians exhibiting lower age-specific incidence rates than non-centenarians is consistent for both men and women (Figures S1 and S2). Smoothing of incidence rates did not alter the overall pattern, and the observed and smoothed rates were consistent (Figure S5, S6). Figure S6 further demonstrates that prostate cancer was rare among centenarians, with almost no cases occurring before age 80.

**Fig. 3:**
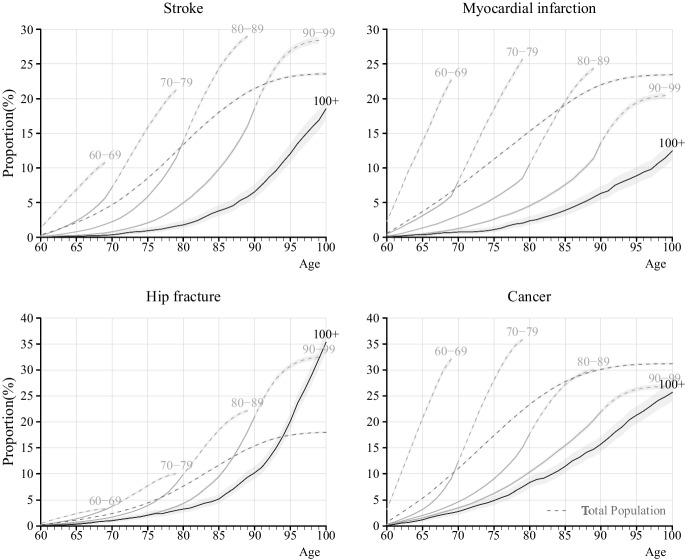
Cumulative incidence of stroke, myocardial infarction, hip fractures, and cancer from age 60 for individuals born between 1912 and 1922, by age at death, in Stockholm County, Sweden. Note: The lines for the cumulative incidence are dashed when part of the individuals in the age-at-death group has died, and solid when conditioning on survival; i.e., in the group that dies between ages 80–89, the line is solid up until age 80, and thereafter dashed. The lightgrey area represents the 95% confidence interval of respective age groups

**Fig. 4:**
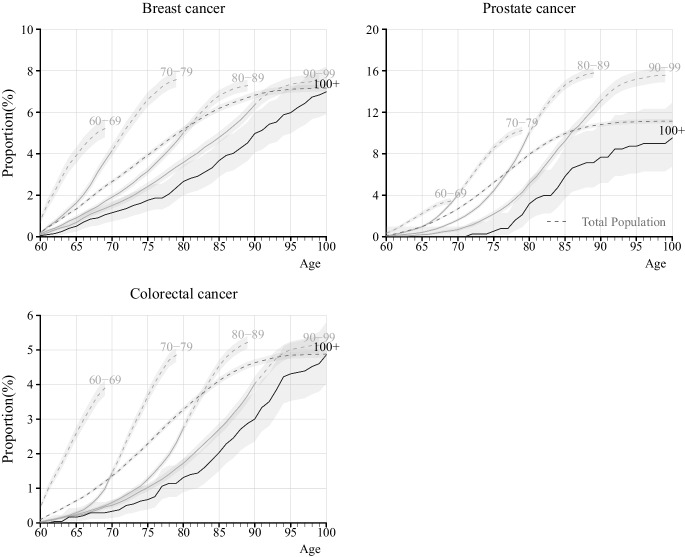
Cumulative incidence of breast, prostate, and colorectal cancer from age 60 for individuals born between 1912 and 1922, by age at death, in Stockholm County, Sweden. Note: The lines for the cumulative incidence are dashed when part of the individuals in the age-at-death group has died, and solid when conditioning on survival; i.e., in the group that dies between ages 80–89, the line is solid up until age 80, and thereafter dashed. The light grey area represents the 95% confidence interval of respective age groups

Figures 3 and 4 present cumulative incidence, or lifetime risk from age 60, of respective disease for the different age-at-death groups. Centenarians exhibited a lower cumulative incidence at every age for all diseases compared to non-centenarians. Moreover, despite living the longest, centenarians had lower lifetime risk of all diseases except hip fractures. In the total population, the lifetime risk of stroke was 23.6%, varying between 10.8% and 29.0% depending on age at death. Centenarians had a lifetime risk of stroke of 18.5%, lower than other age-at-death groups. The lifetime risk of MI was 23.5% in the total population, with a variation of 12.5% for centenarians and 25.6% for those dying in their 70 s. The lifetime risk of hip fracture was 18.0% in the total population, ranging from 3.3% among individuals dying in their 60 s to 35.3% in centenarians. The lifetime risk of cancer was 29.7% in the total population, ranging from 25.7% for centenarians to 35.8% for individuals dying in age 60 s. Individuals dying before age 70 had the lowest lifetime risk of stroke, hip fracture, colorectal cancer, prostate cancer, and breast cancer. The pattern was consistent for both men and women (Figure S3, S4). Centenarians demonstrated lower cumulative incidence at almost every age compared to non-centenarians. However, centenarians had the highest lifetime risk of hip fracture in both men and women.

Finally, we analyzed cumulative incidence of any of the four diseases, shown in Figure S7. This figure reveals that 38.9% of centenarians were free of MI, stroke, cancer, and hip fracture when they turned 100. As a comparison, among individuals dying in the age range of 90–99, the corresponding number is 32.0%. At age 80, 87.5% of centenarians were free of the four diseases, compared to 82.1% among those dying between 90 and 99, and 64.1% among those dying in age range 80–89.

## Discussion

This is the first study to compare disease onset and accumulation between centenarians and non-centenarians at comparable ages earlier in life using longitudinal data. The primary aim was to investigate whether centenarians survive, delay, or avoid diseases to a greater extent than their shorter-lived peers. Our findings show that centenarians have lower age-specific incidence rates at nearly every age and for almost all included diseases compared to those who did not reach age 100, suggesting that centenarians tend to delay disease onset. Furthermore, despite living the longest, centenarians exhibit a lower lifetime risk for all but one of the included diseases. This implies that centenarians not only delay the onset of diseases but also avoid many diseases altogether. These results were consistent across both men and women.

In contrast to the other diseases, centenarians exhibited the highest lifetime risk for hip fractures, approaching 40%. Hip fracture predominantly affects individuals from age 80 onwards [22], with the risk increasing almost exponentially with age. Consistent with our findings, osteoporosis and fractures have been identified as among the most common age-related diseases in supercentenarians (individuals who live to at least 110 years old) in previous studies [25, 26]. Therefore, while the high risk is somewhat anticipated, the extent of the risk is surprisingly significant.

Our findings align with previous evidence indicating that centenarians appear to be relatively healthy [9–11, 14, 25, 27–30]. A Danish study compared the proportion of the population born in 1905 within different age-at-death groups who were free of hospitalization, employing a similar approach to ours [27]. They found that centenarians had a higher proportion of non-hospitalized individuals and fewer hospital days compared to their peers who died at earlier ages. This consistency supports the theory that centenarians delay or even avoid the onset of diseases. A Japanese study investigating supercentenarians in Okinawa observed that the majority experienced few or no major diseases before age 100 [25]. Specifically, coronary heart disease and stroke were rare before age 100, which is also consistent with our results. A Swedish study compared the number of chronic morbidities from age 75 onwards between individuals who survived to age 100 and non-centenarians. They concluded that for chronic diseases, cognitive impairment, and activities of daily living, centenarians had a delayed onset compared to non-centenarians [9]. However, since centenarians lived longer, the study indicated that overall, centenarians spent more years in morbidity than non-centenarians.

The pattern of centenarians having a lower incidence of diseases than non-centenarians at comparable ages earlier in life was similar for both men and women. Few studies have explored sex differences among centenarians and non-centenarians because women typically constitute a dominant proportion of centenarians [9, 31, 32]. However, consistent with our results, the Danish study found that centenarians had a higher proportion of non-hospitalized individuals and fewer hospital days in both men and women. Similarly, a Spanish study exploring sex differences among centenarians, octogenarians, and nonagenarians observed a lower prevalence of cerebrovascular disease, MI, and cancer among both male and female centenarians compared to octogenarians and nonagenarians [33]. These findings align with our results.

Even if our study does not explore the mechanisms to why centenarians delay and avoid diseases, rather than survive them to a higher extent, it opens for some speculation of these. The finding that centenarians avoid cardiovascular diseases and never reach the level of non-centenarians suggests that they are resilient to these diseases. This resilience could be related both to lifestyle factors [34] and genetic factors [35]. A previous study comparing biomarker profiles of centenarians and non-centenarians earlier in life indeed found centenarians had healthier values of biomarkers related to cardiovascular diseases, for example glucose, lipids, and uric acid [28]. As for genetic factors, some previous studies have tried to elucidate the mechanism of disease development among centenarians using whole genome sequencing [10, 36]. However, they have found that centenarians have as many disease-related genes as non-centenarians, and yet they avoid the diseases. Whether this depends on gene-environmental interaction [37] or the presence of longevity-associated genetic variants that offset the negative effects of the mutations remains unknown [10, 38]. There have been numerous candidate gene studies and Genome-wide Association Study (GWAS) studies, including individuals of diverse ancestry, which have explored longevity associated genes [35, 36, 38–41]. The only consensus seems to be regarding APOE [39, 42]. The APOE ε2 and ε4 variants are associated with a decreased (ε2) or increased (ε4) risk for several age-related diseases and associated with longevity [40, 41]. Hopefully, future studies can continue to explore how and why longevity-associated variants are triggered in centenarians but not in individuals dying earlier, and how certain lifestyle factors contribute to the discrepancy in genetic variants. We also need to better understand when in the life course, lifestyle and genetic factors play the biggest role, and how their impact changes over time.

Hip fracture was an exception in terms of avoidance of disease. Even if centenarians had lower age-specific incidence of hip fractures, they attained it eventually. The etiology of hip fracture is multidimensional and relies on both lifestyle and genetic factors [43, 44]. Bone mass and density decline with age. It seems that this process is somewhat delayed in centenarians, but perhaps there is a threshold, at least now, for when this process cannot be postponed anymore. It may also be the case that preventive strategies for osteoporosis, falls, and fractures need be improved among the very oldest.

### Strengths and limitations

Intuitively, it may seem a bit odd to condition on death. But when examining the life course of individuals who survive to the age of 100, it is a strength to do so for individuals born in the same year. It prevents distortions from period and cohort effects. Moreover, it may seem obvious that individuals who survive to age 100 are healthier at younger ages than those who are about to die. However, what we observe is that centenarians are healthier compared to all other individuals. Already at age 70, they differ from individuals living to age 90. Moreover, we show that at the age of 100, they have still not caught up with the disease risks that individuals who die at earlier ages do. This adds to the evidence that longer life expectancy is not accompanied by higher rates of disease or that diseases simply move up in age. Rather, it points to centenarians being a group of people who manage to avoid age-related diseases, especially cardiovascular diseases. The possibility to identify all individuals in the population and their disease history prevents selection bias.

Although individuals dying at different ages were compared at the same ages regarding disease incidence, improvements in disease detection over time could have influenced our results. For example, increased awareness and knowledge and improved diagnostics and detection may have increased the incidence of some diseases over time, perhaps most notably cancer. It would mean that individuals reaching older ages are more likely to be diagnosed than individuals at younger ages in the past. Nevertheless, we found consistently lower incidence rates for individuals dying at older ages. In the case of cardiovascular diseases, it is more complex. While disease detection has increased over time as for cancer, also secondary prevention such as hypertension treatment, lipid-lowering treatment of low-density lipoprotein cholesterol and the introduction of more potent drugs have increased over time. Blood pressure control in hypertensive patients attending Swedish primary care has improved over the period from 2001 to 2007, which may partially be due to updates of drug classes used and the use of more effective drug combinations [45]. High-intensity lipid-lowering therapy became more frequent in Sweden over the past decade, consistent with the updated treatment recommendations by the Swedish Medical Product Agency in 2014 and European Society of Cardiology guidelines published after 2010 [46]. These advances have likely prevented some strokes and MIs, contributing to the lower incidence rates among those aged 90 + and centenarians compared to a comparable earlier period. However, for example, previous research found that diuretics were more frequently used in centenarians, while newer types of cardiovascular drugs were less common compared to those dying earlier [12, 47]. This suggests that centenarians are not prescribed cardiovascular drugs to the same extent as younger elderly adults, and there is often hesitation to introduce new drugs to very old patients [47]. Moreover, the incidence rates at comparable ages—such as age 70, 75, and 80—were subject to the same conditions for all groups since they were from the same birth cohorts and estimated at the same time.

Finally, it should be noted that our results and conclusions are based on reaching age 100. After age 100, centenarians may develop some of the included diseases, resulting in a higher lifetime risk than observed up until age 100. While it appears that centenarians delay or avoid many chronic conditions until age 100, it is intriguing to explore their health panorama beyond that milestone, including the diseases they may develop and the causes of their deaths. However, capturing the cause of death for centenarians is complex, as the causes are often unknown and difficult to determine [11, 48].

In conclusion, this comprehensive study shows that centenarians exhibit lower age-specific disease incidence rates and lower lifetime risks of major diseases compared to individuals who do not reach 100 years of age. Across multiple diseases including stroke, myocardial infarction, and various cancers, centenarians consistently demonstrated the lowest age-specific incidence rates, highlighting their ability to delay and even avoid these chronic conditions until very late in life. The exception was hip fracture where incidence rose sharply right before age 100 and centenarians exhibited the highest lifetime risk. Overall, the findings challenge the notion that longer life expectancy inevitably leads to higher disease rates or a simple shift of diseases to older ages. Instead, they suggest that centenarians constitute a distinct group with exceptional health resilience to diseases, capable of effectively avoiding age-related diseases, particularly cardiovascular diseases. Future research should continue to compare also less severe health conditions for centenarians and non-centenarians to increase the understanding of the pathways to healthy longevity.

## Supplementary Information

Below is the link to the electronic supplementary material.

Supplementary file1 (PDF 1275 KB)

## Data Availability

The individual level data underlying this study cannot be shared publicly because of the General Data Protection Regulation in Sweden. Access to the data and statistical code can be permitted to external researchers after ethical vetting and establishment of a collaboration agreement. Contact the corresponding author for questions about data sharing.
