# Supplementary material for: Do people reach 100 by surviving, delaying, or avoiding diseases? A life course comparison of centenarians and non-centenarians from the same birth cohorts
Source: GeroScience. 2024 Aug 30;47(3):3539–49. doi: 10.1007/s11357-024-01330-w (PMC12181509; doi:10.1007/s11357-024-01330-w)
Supplement: Supplementary file 1 — Supplementary file1 (PDF 1275 KB) [file 11357_2024_1330_MOESM1_ESM.pdf]

Supplementary materials

Tables

Table S1 Characteristics of women, total and stratified by age at death, aged 60 years and above in Stockholm County, Sweden, 1972-2022

Table S2 Characteristics of men, total and stratified by age at death, aged 60 years and above in Stockholm County, Sweden, 1972-2022

Table S3 Characteristics of individuals with respective diseases, aged 60 years and above, in Stockholm County, Sweden,1972-2022

Table S4 Characteristics of women with respective diseases, aged 60 years and above, in Stockholm County, Sweden, 1972-2022

Table S5 Characteristics of men with respective diseases, aged 60 years and above, in Stockholm County, Sweden, 1972-2022

Figures

Figure S1 Age-specific incidence rates of stroke, myocardial infarction, hip fractures and cancer from age 60 for men and women born between 1912-1922, by age at death, in Stockholm County, Sweden, 1972-2022

Figure S2 Age-specific incidence rates of breast, prostate and colorectal cancer from age 60 for men and women born between 1912-1922, by age at death, in Stockholm County, Sweden, 1972-2022

Figure S3 Cumulative incidence of stroke, myocardial infarction, hip fractures and cancer from age 60 for men and women born between 1912-1922, by age at death, in Stockholm County, Sweden, 1972-2022

Figure S4 Cumulative incidence of breast, prostate and colorectal cancer from age 60 for men and women born between 1912-1922, by age at death, in Stockholm County, Sweden, 1972-2022

Figure S5 Observed and smoothed age-specific incidence rates of stroke, myocardial infarction, hip fractures and cancer from age 60 for individuals born between 1912-1922, by age at death, in Stockholm County, Sweden, 1972-2022

Figure S6 Observed and smoothed age-specific incidence rates of breast, prostate and colorectal cancer from age 60 for individuals born between 1912-1922, by age at death, in Stockholm County, Sweden, 1972-2022

Figure S7 Cumulative incidence of any of the four diseases (stroke, myocardial infarction, hip fracture, and cancer) from age 60 for individuals born between 1912-1922, by age at death, in Stockholm County, Sweden, 1972-2022

Table S1 Characteristics of women, total and stratified by age at death, aged 60 years and above in Stockholm County, Sweden, 1972-2022

	Died between ages 60-69 years	Died between ages 70-79 years	Died between ages 80-89 years	Died between ages 90-99 years	Died >=100 years	Total	Age at diagnosis, median (IQR [a])
No. of individuals, (%)	10,120 (11.2)	20,050 (22.2)	33,990 (37.7)	24,019 (26.6)	1989 (2.3)	90,168	
No. of patients, (%)							
Stroke	1097 (10.8)	4244 (21.2)	9851 (29.0)	6725 (28.0)	354 (17.8)	22,271 (24.7)	81.0 (75.0-87.0)
Myocardial infarction	1528 (15.1)	4160 (20.8)	7094 (20.9)	4356 (18.1)	216 (10.9)	17,354 (19.2)	80.0 (73.0-86.0)
Hip fracture	418 (4.1)	2707 (13.5)	9470 (27.9)	8882 (37.0)	748 (37.6)	22,225 (24.6)	83.0 (76.0-88.0)
All cancer	3752 (37.1)	7128 (35.6)	8764 (25.8)	5585 (23.3)	474 (23.8)	25,703 (28.5)	75.0 (68.0-82.0)
Colorectal cancer	509 (5.0)	1017 (5.1)	1622 (4.8)	1142 (4.8)	94 (4.7)	4384 (4.9)	77.0 (70.5-84.0)
Breast cancer	528 (5.2)	1520 (7.6)	2478 (7.3)	1798 (7.5)	139 (7.0)	6463 (7.2)	74.0 (67.0-81.0)

[a] IQR= interquartile range

Table S2 Characteristics of men, total and stratified by age at death, aged 60 years and above in Stockholm County, Sweden, 1972-2022

	Died between ages 60-69 years	Died between ages 70-79 years	Died between ages 80-89 years	Died between ages 90-99 years	Died >=100 years	Total	Age at diagnosis, median (IQR [a])
No. of individuals, (%)	18,367 (22.8)	25,826 (32.0)	25,840 (32.1)	10,208 (12.7)	378 (0.5)	80,619	
No. of patients, (%)							
Stroke	1966 (10.7)	5496 (21.3)	7478 (29.0)	2998 (29.4)	85 (22.5)	18,023 (22.4)	76.0 (70.0-82.0)
Myocardial infarction	4934 (26.9)	7602 (29.4)	7466 (28.9)	2641 (25.9)	79 (20.9)	22,722 (28.2)	73.0 (66.0-80.0)
Hip fracture	515 (2.8)	1903 (7.4)	3777 (14.6)	2215 (21.7)	88 (23.3)	8498 (10.5)	81.0 (73.0-87.0)
All cancer	5384 (29.3)	9311 (36.1)	9188 (35.6)	3590 (35.2)	134 (35.4)	27,607 (34.2)	74.0 (68.0-80.0)
Colorectal cancer	601 (3.3)	1209 (4.7)	1502 (5.8)	616 (6.0)	21 (5.6)	3949 (4.9)	75.0 (69.0-81.0)
Prostate cancer	631 (3.4)	2649 (10.3)	4081 (15.8)	1590 (15.6)	36 (9.5)	8987 (11.2)	76.0 (71.0-81.0)

[a] IQR= interquartile range

Table S3 Characteristics of individuals with respective diseases, aged 60 years and above, in Stockholm County, Sweden,1972-2022

	Stroke	Myocardial infarction	Hip fracture	Cancer			
				Any site	Colorectal	Breast	Prostate
No. of patients	40,294	40,076	30,723	53,310	8333	6463	8987
Age at diagnosis, N (%)							
60-69	6969 (17.3)	11,188 (27.9)	3572 (11.6)	16,810 (31.5)	2026 (24.3)	2186 (33.8)	1849 (20.6)
70-79	14,177 (35.2)	13,578 (33.9)	8182 (26.6)	21,190 (39.7)	3255 (39.0)	2272 (35.1)	4104 (45.7)
80-89	14,606 (36.2)	12,057 (30.1)	13,444 (43.8)	12,854 (24.1)	2507 (30.1)	1595 (24.7)	2689 (29.9)
90-99	4503 (11.2)	3229 (8.1)	5450 (17.7)	2436 (4.6)	539 (6.5)	407 (6.3)	343 (3.8)
100 and above	39 (0.1)	24 (0.1)	75 (0.2)	20 (0.0)	6 (0.1)	3 (0.0)	2 (0.0)
No. of incident events	56,776	53,869	32,367	55,390	8703	6718	9344

Table S4 Characteristics of women with respective diseases, aged 60 years and above, in Stockholm County, Sweden,1972-2022

	Stroke	Myocardial infarction	Hip fracture	Cancer		
				Any site	Colorectal	Breast
No. of patients	22,271	17,354	22,225	25,703	4384	6463
Age at diagnosis, N (%)						
60-69	2627 (11.8)	2881 (16.6)	2185 (9.8)	7893 (30.7)	960 (21.9)	2186 (33.8)
70-79	7112 (31.9)	5515 (31.8)	5784 (26.0)	9543(37.1)	1599 (36.5)	2272 (35.1)
80-89	9131 (41.0)	6694 (38.6)	10,021 (45.1)	6628 (25.8)	1448 (33.0)	1595 (24.7)
90-99	3370 (15.1)	2243 (12.9)	4175 (18.8)	1622 (6.3)	373(8.5)	407 (6.3)
100 and above	31 (0.1)	21 (0.1)	60 (0.3)	17 (0.1)	4 (0.1)	3 (0.0)
No. of incident events	31,151	23,133	23,451	26,801	4593	6718

Table S5 Characteristics of men with respective diseases, aged 60 years and above, in Stockholm County, Sweden,1972-2022

	Stroke	Myocardial infarction	Hip fracture	Cancer		
				Any site	Colorectal	Prostate
No. of patients	18,023	22,722	8498	27,607	3949	8987
Age at diagnosis, N (%)						
60-69	4342 (24.1)	8307 (36.6)	1387 (16.3)	8917 (32.3)	1066 (27.0)	1849 (20.6)
70-79	7065 (39.2)	8063 (35.5)	2398 (28.2)	11,647 (42.2)	1656 (41.9)	4104 (45.7)
80-89	5475 (30.4)	5363 (23.6)	3423 (40.3)	6226 (22.6)	1059 (26.8)	2689 (29.9)
90-99	1133 (6.3)	986 (4.3)	1275 (15.0)	814 (2.9)	166 (4.2)	343 (3.8)
100 and above	8 (0.0)	3 (0.0)	15 (0.2)	3 (0.0)	2 (0.1)	2 (0.0)
No. of incident events	25,625	30,741	8916	28,589	4110	9344

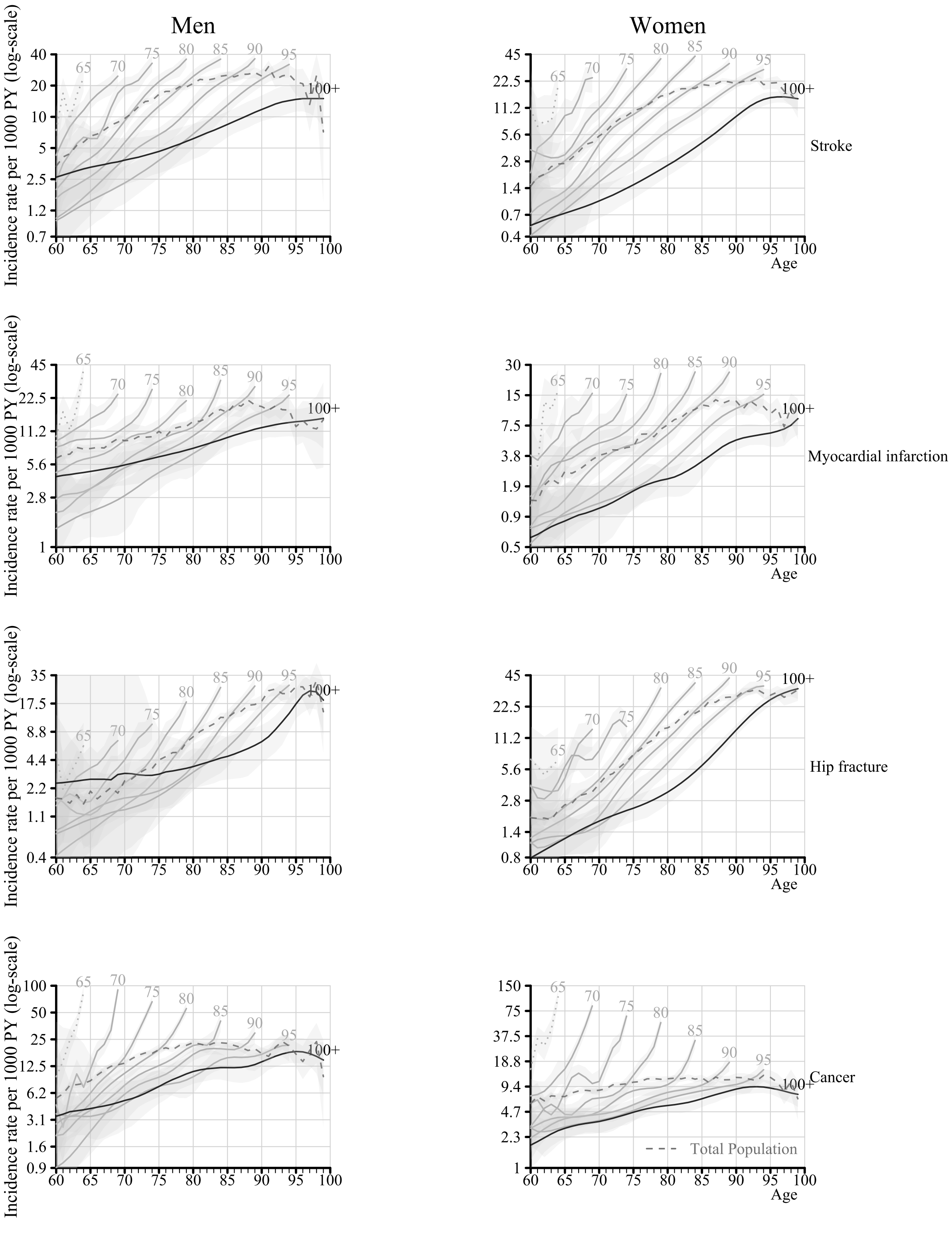

Figure S1 Age-specific incidence rates of stroke, myocardial infarction, hip fractures and cancer from age 60 for men and women born between 1912-1922, by age at death, in Stockholm County, Sweden, 1972-2022

Notes: Solid lines represent the smoothed rates while dashed line for age 65 represents the observed rates. The lightgrey area represents the 95% confidence interval. The x-axis represents the chronological ages of everyone followed from age 60 until death or becoming centenarians. The numbers by each line represent age at death.

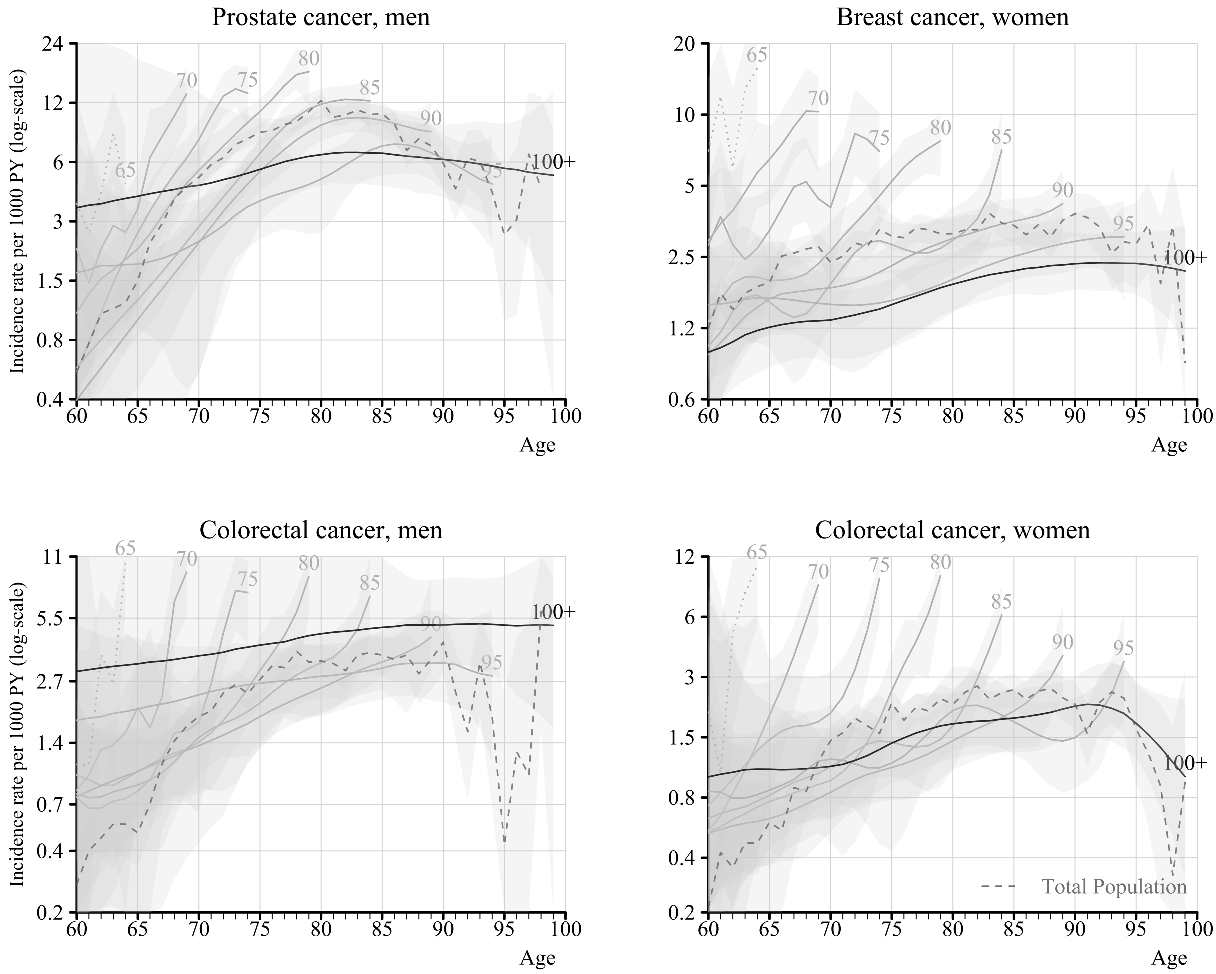

Figure S2 Age-specific incidence rates of breast, prostate and colorectal cancer from age 60 for men and women born between 1912-1922, by age at death, in Stockholm County, Sweden, 1972-2022

Notes: Solid lines represent the smoothed rates while dashed line for age 65 represents the observed rates. The lightgrey area represents the 95% confidence interval. The x-axis represents the chronological ages of each participant followed from age 60 until death or becoming centenarians. The numbers by each line represent age at death.

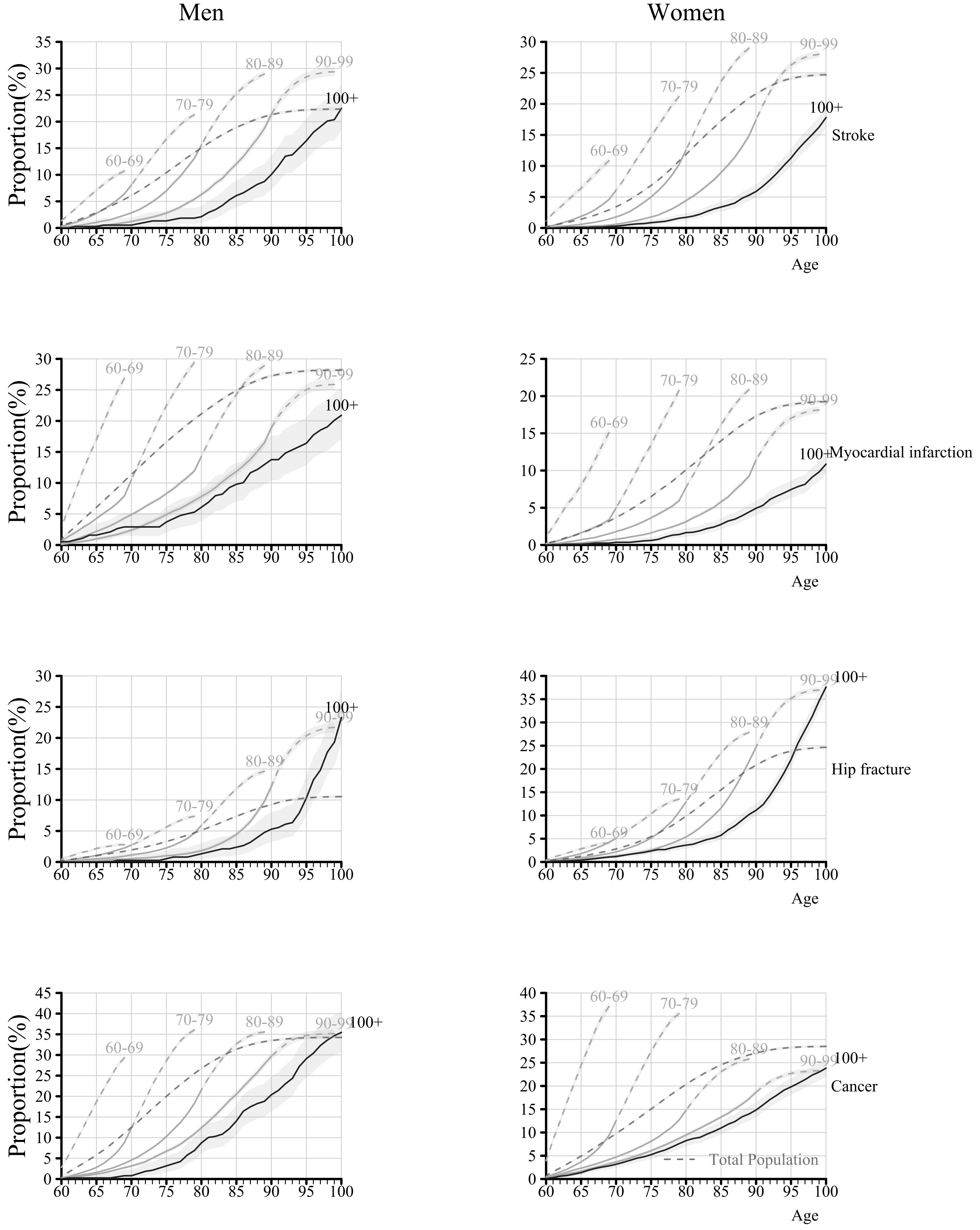

Figure S3 Cumulative incidence of stroke, myocardial infarction, hip fractures and cancer from age 60 for men and women born between 1912-1922, by age at death, in Stockholm County, Sweden, 1972-2022

Note: The lines for the cumulative incidence are dashed when part of the individuals in the age-at-death group has died, and solid when conditioning on survival, i.e in the group that dies between ages 80-89 the line is solid up until age 80, and thereafter dashed. The lightgrey area represents the 95% confidence interval of respective age groups.

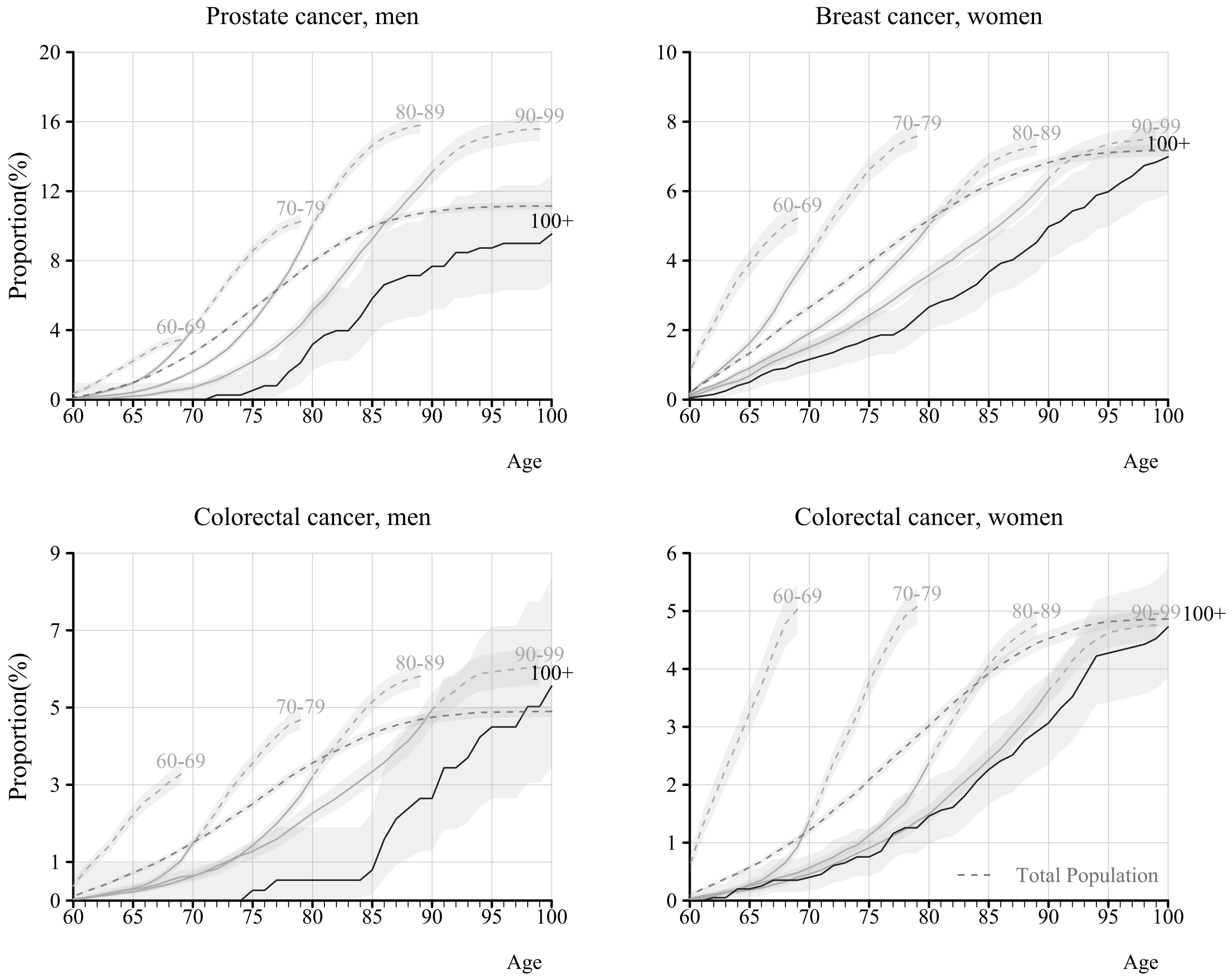

Figure S4 Cumulative incidence of breast, prostate and colorectal cancer from age 60 for men and women born between 1912-1922, by age at death, in Stockholm County, Sweden, 1972-2022

Note: The lines for the cumulative incidence are dashed when part of the individuals in the age-at-death group has died, and solid when conditioning on survival, i.e in the group that dies between ages 80-89 the line is solid up until age 80, and thereafter dashed. The lightgrey area represents the 95% confidence interval of respective age groups.

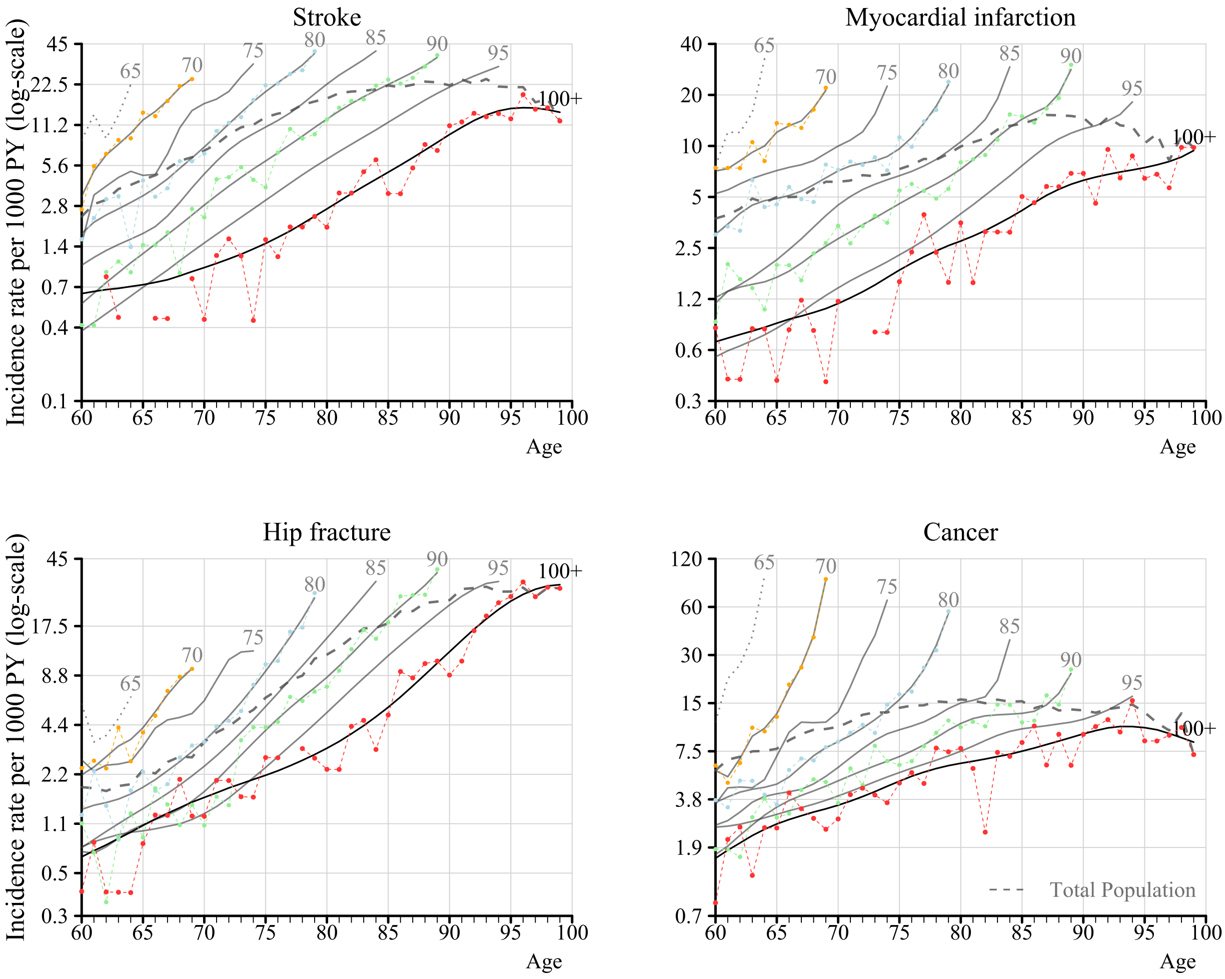

Figure S5 Observed and smoothed age-specific incidence rates of stroke, myocardial infarction, hip fractures and cancer from age 60 for individuals born between 1912-1922, by age at death, in Stockholm County, Sweden, 1972-2022

Notes: Solid lines represent the smoothed rates for centenarians and non-centenarians. Dashed line for age 65 represents the observed rates. Observed incidence rates for those died at age 70 (orange dotted line), age 80 (light-blue dotted line), age 90 (light-green dotted line) and as centenarians (red dotted line) are also presented to show the consistency between smoothed and observed rates. The x-axis represents the chronological ages of everyone followed from age 60 until death or becoming centenarians. The numbers by each line represent age at death.

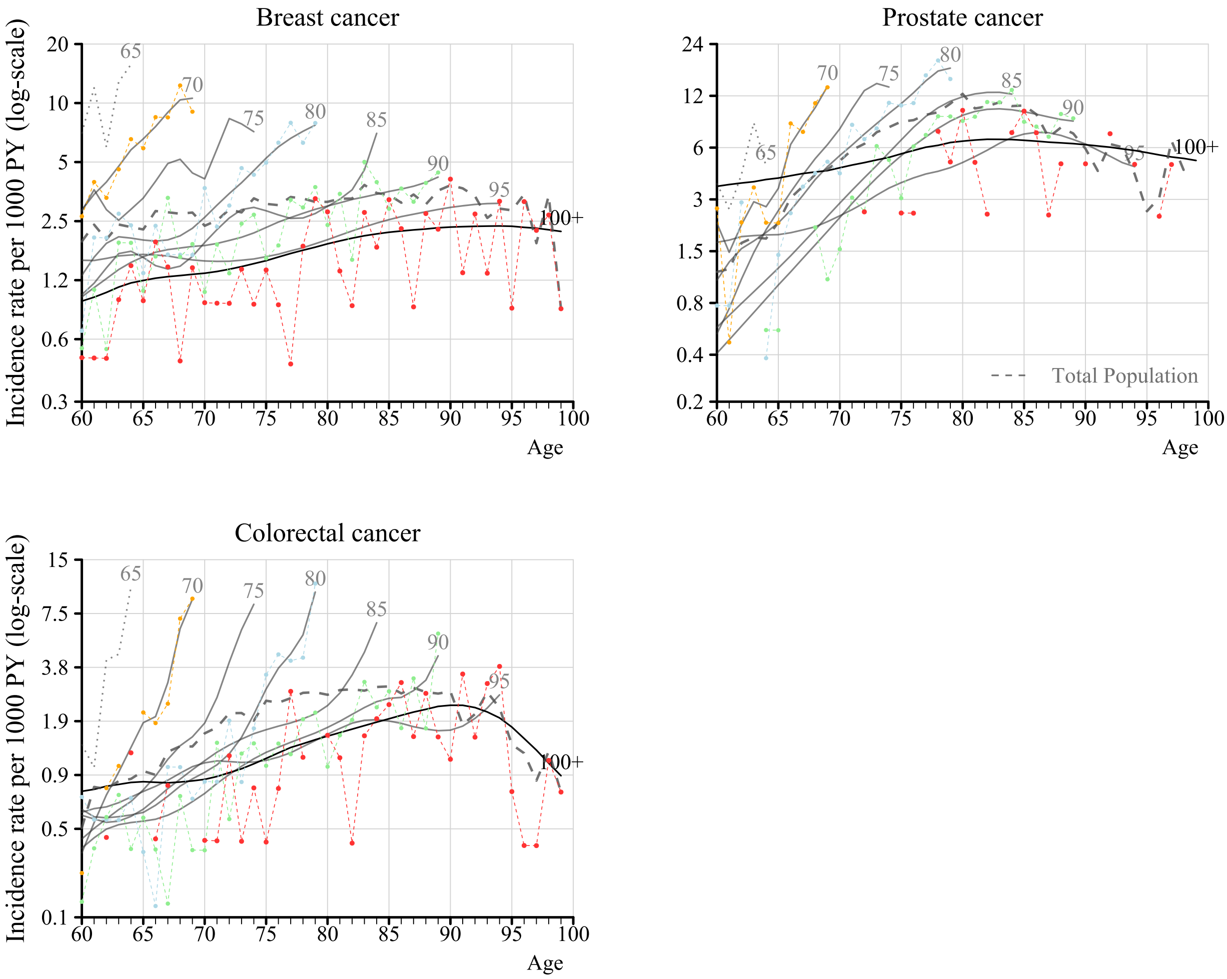

Figure S6 Observed and smoothed age-specific incidence rates of breast, prostate and colorectal cancer from age 60 for individuals born between 1912-1922, by age at death, in Stockholm County, Sweden, 1972-2022

Notes: Solid lines represent the smoothed rates for centenarians and non-centenarians. Dashed line for age 65 represents the observed rates. Observed incidence rates for those died at age 70 (orange dotted line), age 80 (light-blue dotted line), age 90 (light-green dotted line) and as centenarians (red dotted line) are also presented to show the consistency between smoothed and observed rates. The x-axis represents the chronological ages of everyone followed from age 60 until death or becoming centenarians. The numbers by each line represent age at death.

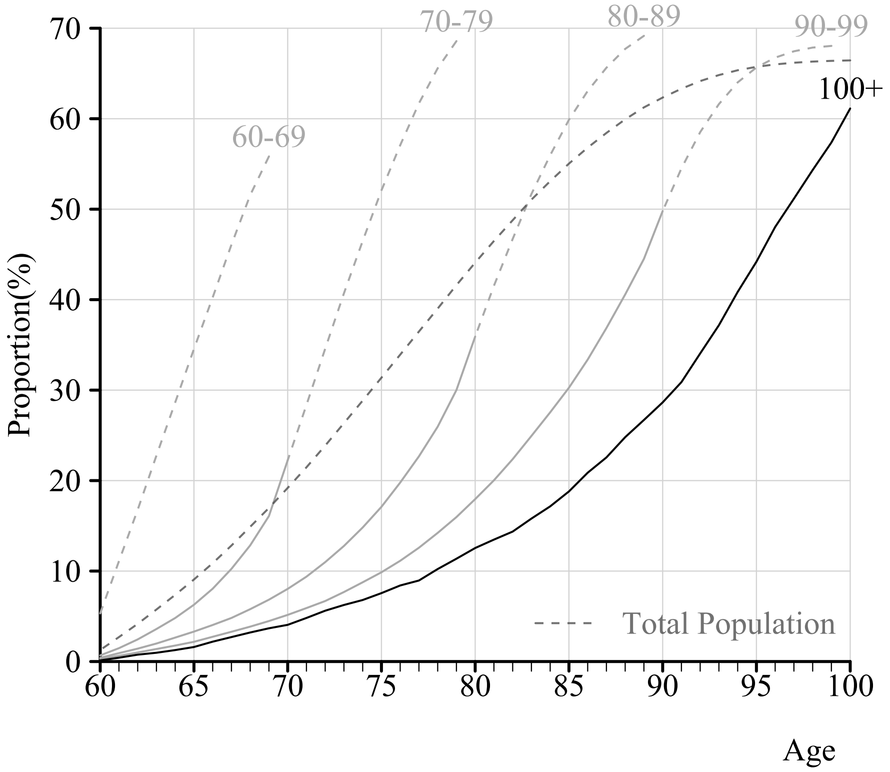

Figure S7 Cumulative incidence of any of the four diseases (stroke, myocardial infarction, hip fracture, and cancer) from age 60 for individuals born between 1912-1922, by age at death, in Stockholm County, Sweden, 1972-2022

Notes: The lines for the cumulative incidence are dashed when part of the individuals in the age-at-death group has died, and solid when conditioning on survival, i.e in the group that dies between ages 80-89 the line is solid up until age 80, and thereafter dashed.
